# Noise reduction in dual‐energy computed tomography virtual monoenergetic imaging

**DOI:** 10.1002/acm2.12694

**Published:** 2019-08-07

**Authors:** Chi‐Kuang Liu, Hsuan‐Ming Huang

**Affiliations:** ^1^ Department of Medical Imaging Changhua Christian Hospital Changhua City Taiwan; ^2^ Institute of Medical Device and Imaging, College of Medicine National Taiwan University Taipei Taiwan

**Keywords:** dual‐energy computed tomography, noise reduction, virtual monoenergetic images

## Abstract

**Purpose:**

Virtual monoenergetic images (VMIs) derived from dual‐energy computed tomography (DECT) have been explored for several clinical applications in recent years. However, VMIs at low and high keVs have high levels of noise. The aim of this study was to reduce image noise in VMIs by using a two‐step noise reduction technique.

**Methods:**

VMI was first denoised using a modified highly constrained backprojection (HYPR) method. After the first‐step denoising, a general‐threshold filtering method was performed. Two sets of anthropomorphic phantoms were scanned with a clinical dual‐source DECT system. DECT data (80/140Sn kV) were reconstructed as VMI series at 12 different energy levels (range, 40‐150 keV, interval, 10 keV). For comparison, the averaged VMIs obtained from 10 repeated DECT scans were used as the reference standard. The signal‐to‐noise ratio (SNR), contrast‐to‐noise ratio (CNR) and root‐mean‐square error (RMSE) were used to evaluate the quality of VMIs.

**Results:**

Compared to the original HYPR method, the proposed two‐step image denoising method could provide better performance in terms of SNR, CNR, and RMSE. In addition, the proposed method could achieve effective noise reduction while preserving edges and small structures, especially for low‐keV VMIs.

**Conclusion:**

The proposed two‐step image denoising method is a feasible method for reducing noise in VMIs obtained from a clinical DECT scanner. The proposed method can also reduce edge blurring and the loss of intensity in small lesions.

## 1 INTRODUCTION

After introduction of the first commercial dual‐source dual‐energy computed tomography (DECT) system in 2006,^1^ several DECT‐based techniques including iodine map, virtual non‐contrast and effective atomic number have been proposed.^2^, ^3^ These DECT‐based techniques offer a wide variety of clinical applications.^2^, ^3^ In particular, virtual monoenergetic images (VMIs) derived from DECT images have shown encouraging results and gained popularity recently.^4^ Clinical applications of DECT‐based VMIs include metal artifact reduction, beam‐hardening correction,^5^, ^6^ contrast and noise optimization,^7^, ^8^ and material differentiation.^9^, ^10^ In addition, DECT‐based VMIs can be used to assess fatty liver^11^ and hypervascularized abdominal tumors.^12^ Despite promising results obtained in recent investigations, the noise levels of DECT‐based VMIs were high at low and high keVs.^7^, ^8^, ^13^, ^14^

Many different strategies have been developed to reduce image noise in DECT‐based VMIs. For example, VMIs obtained from either projection‐ or image‐based methods can be improved using iterative optimization algorithms. Several previous studies demonstrated the feasibility of using iterative optimization algorithms to improve the image quality of DECT and resulting materials decomposition.^15^, ^16^ Consequently, the reconstruction of DECT‐based VMIs can be improved. However, these advanced optimization methods are not provided by scanner vendors. Moreover, the raw data format is not explicitly described by scanner vendors, and image reconstruction requires calibration data. Lack of access to this data makes it difficult to implement the reconstruction of VMIs. One possible method to improve the image quality of VMIs is to apply an image denoising method directly on the DECT‐derived VMIs.^14^ One previous study showed that the highly constrained backprojection (HYPR) method^17^, ^18^ can be exploited to reduce noise in photon counting‐based monoenergetic images.^13^ In addition, HYPR has been used for reducing image noise in dynamic contrast‐enhanced perfusion CT.^19^, ^20^

Although post‐reconstruction denoising can improve the quality of monoenergetic images obtained from a preclinical photon counting‐based spectral CT scanner,^13^ it has not been validated using clinical DECT‐derived VMIs. Moreover, reducing the radiation dose from DECT remains an important topic.^21^ Using a low‐dose DECT scan would increase the noise level which may affect the performance of HYPR. In order to further improve the image quality of DECT‐based VMIs, we proposed a two‐step noise reduction technique using a combination of HYPR^17^, ^18^ and the general‐threshold filtering (GTF) method.^22^ In this study, the proposed two‐step noise reduction method was compared with the original HYPR method.^17^, ^18^ Two sets of anthropomorphic phantoms were used to assess the image quality and signal characteristics of denoised VMIs. We also investigated whether the studied image denoising methods could effectively reduce image noise while preserving edges and small structures.

## 2 MATERIALS AND METHODS

### 2.1 Two‐step noise reduction technique

In this study, we propose a two‐step noise reduction technique to reduce image noise in DECT‐derived VMIs by using a combination of HYPR^17^, ^18^ and GTF.^22^ Firstly, VMIs obtained from vendor software were denoised using HYPR.^17^, ^18^ As originally developed for contrast‐enhanced magnetic resonance angiography,^17^ HYPR is a postprocessing technique that uses information obtained from all time‐series images to improve the image quality of each individual time‐series image. In brief, the HYPR‐processed VMI (V^HYPR^) at a virtual monochromatic energy level denoted by E is calculated as follows:

(1) $${\text{V}}^{\text{HYPR}}\left(\text{E}\right)=\text{CI}\times \frac{\text{F}⊗\text{V}\left(\text{E}\right)}{\text{F}⊗\text{CI}}$$

where V(E) is the VMI at the energy level of E (keV) obtained from vendor software, CI is the composite image obtained from the sum of 12 V(E) (i.e. 40 to 150 keV in 10 keV intervals) and F is a box‐kernel (low‐pass) spatial filter function.^17^, ^18^ A 7 × 7 pixel uniform square kernel used in two previous studies^13^, ^20^ can effectively reduce image noise, though it may lead to edge blurring and loss of intensity in small lesions. To resolve this problem, we modified HYPR to use a two‐dimensional adaptive noise‐removal (Wiener) filter^23^ instead of a uniform square convolution kernel. Moreover, to balance noise removal and edge preservation, we used results obtained from the adaptive Wiener filter with two different window sizes (i.e. 3 × 3 and 7 × 7). The final formula of the modified HYPR is:

(2) $${\text{V}}^{\text{mHYPR}}\left(\text{E}\right)=0.5\times \text{CI}\times \left[\frac{{\text{WF}}_{3\times 3}\left(\text{V}\left(\text{E}\right)\right)}{{\text{WF}}_{3\times 3}\left(\text{CI}\right)}+\frac{{\text{WF}}_{7\times 7}\left(\text{V}\left(\text{E}\right)\right)}{{\text{WF}}_{7\times 7}\left(\text{CI}\right)}\right]$$

where ${\text{WF}}_{\text{n}\times \text{n}}\left(\text{V}\left(\text{E}\right)\right)$ denotes the adaptive Wiener filtering of V(E) with n × n window size. Note that the Wiener filter requires the noise variance to be set to the average of all the local estimated variances.

After the first‐step processing, we observed that the modified HYPR reduced image noise only moderately. Thus, the HYPR‐processed VMI obtained from Eq. (2) was refined by a second step. This second step used a GTF method originally developed for CT image reconstruction.^24^ Our previous study demonstrates that the GTF method can be used for denoising diffusion weighted magnetic resonance imaging,^22^ and it has good edge‐preserving smoothing property.^22^ In brief, GTF applied to V^mHYPR^ (E) can be described as follows^22^:

(3) $${\text{V}}_{\text{i}}^{\text{GTF}}\left(\text{E}\right)=\frac{1}{4}\sum_{{\text{i}}^{\prime }}\text{Q}\left({\text{V}}_{\text{i}}^{\text{mHYPR}}\left(\text{E}\right),{\text{V}}_{{\text{i}}^{\prime }}^{\text{mHYPR}}\left(\text{E}\right),\lambda ,\text{p}\right),\;{\text{i}}^{\prime }\in \;{\text{N}}_{\text{i}}$$

and

(4) $$\text{Q}\left({\text{V}}_{1},{\text{V}}_{2},\lambda ,\text{p}\right)=\left\{\begin{array}{cc} \left({\text{V}}_{1}+{\text{V}}_{2}\right)/2 & \text{if}\;\left|{\text{V}}_{1}-{\text{V}}_{2}\right|<\omega_{\lambda ,\text{p}}\\ {\text{V}}_{1}-\text{sgn}\left({\text{V}}_{1}-{\text{V}}_{2}\right)\times {\text{h}}_{\lambda ,\text{p}}\left({\text{V}}_{1},{\text{V}}_{2}\right)/2 & \text{if}\;\left|{\text{V}}_{1}-{\text{V}}_{2}\right|\geq \omega_{\lambda ,\text{p}} \end{array}\right.$$

where ${\text{h}}_{\lambda ,\text{p}}\left({\text{V}}_{1},{\text{V}}_{2}\right)=0.5\times \lambda \times \text{p}\times {\left\{\left[\left|{\text{V}}_{1}-{\text{V}}_{2}\right|-0.5\times \lambda \times \text{p}\right]\times {\left[\left|{\text{V}}_{1}-{\text{V}}_{2}\right|-0.5\times \text{p}\times \text{pp}\right]}^{\text{p}-1}\right\}}^{\text{p}-1}$, $\omega_{\lambda ,\text{p}}=0.5\times \left(2\;\text{- p}\right)\times \text{pp}$, $\text{pp}=\left[{\left(1-\text{p}\right)}^{\left(\text{p}-1\right)/\left(2-\text{p}\right)}\right]\times \left[\lambda^{1/\left(2-\text{p}\right)}\right]$, N_i_ represents the 4‐neighborhood of the i^th^ pixel, λ is the regularization parameter that controls the filtering strength and p (= 0.9) is the norm of the regularization term. Further details on the GTF method can be found in Refs. [22,24]. In this study, λ was set to the noise level of V^mHYPR^ (E) obtained from the method described in Ref. [25]. The filtering process shown in Eq. (3) was repeated 40 times in order to obtain sufficient noise reduction.

Although GTF can remove noise while preserving edges, it leads to a certain loss of intensity in edges and small lesions. To address this problem, an additional step was performed to recover the intensity of edges and small lesions. First, we applied the Canny's edge detection algorithm to V^GTF^ (E). Second, the edge (binary) image was dilated using a disk shaped structuring element with radius of 2 pixels. The dilated edge image may contain many pixels which had signal loss. Finally, the average of V^GTF^ (E) and V^mHYPR^ (E) was assigned to pixels belonging to the edges. Since V^mHYPR^ (E) was less blurred than V^GTF^ (E), the average of V^GTF^ (E), and V^mHYPR^ (E) can alleviate the loss of intensity while maintaining image quality. The final denoised $\text{VMI}\left({\text{V}}_{\text{i}}^{\text{F}}\left(\text{E}\right)\right)$ would be:

(5) $${\text{V}}_{\text{i}}^{\text{F}}\left(\text{E}\right)=\left\{\begin{array}{cc} \left[{\text{V}}_{\text{i}}^{\text{mHYPR}}\left(\text{E}\right)+{\text{V}}_{\text{i}}^{\text{GTF}}\left(\text{E}\right)\right]/2 & \text{if}\;\text{i}\in \text{edges}\\ {\text{V}}_{\text{i}}^{\text{GTF}}\left(\text{E}\right) & \text{otherwise} \end{array}\right.$$

Note that VMIs read from DICOM files have pixel values ranging from 0 to 4095. After noise reduction, all processed VMIs were subtracted by 1024 to yield Hounsfield units (HU).

### 2.2 Phantoms, DECT data acquisition, and image reconstruction

Two sets of anthropomorphic phantoms were scanned using a second generation dual source CT scanner (SOMATOM Definition Flash, Siemens Healthcare, Forchheim, Germany). For both phantoms, DECT data were acquired using a DE default scan protocol at 80 kV*/*Sn140 kV, 200*/*95 effective mAs. Other settings were: gantry rotation time, 0.5 s; pitch, 0.6 and collimation, 32 mm × 0.6 mm. All DECT raw data were reconstructed with a dedicated dual‐energy filtered back projection medium‐soft convolution kernel (D30f). DECT image series were exported as axial images with a slice thickness of 1.5 mm and an increment of 1 mm. Finally, VMIs from 40 to 150 keV in 10 keV intervals were reconstructed with a dedicated application (Monoenergetic Application Class) and software on a multimodality workstation (Syngo MMWP VE 40A, Siemens Healthcare, Forchheim, Germany).

### 2.3 Data analysis

In this study, averaged VMIs obtained from 10 repeated DECT scans were used as reference standards. The averaged VMIs have higher image quality than conventional VMIs obtained from one normal‐dose (ND) DECT scan. For objective comparison of different VMIs, we calculated the signal‐to‐noise ratio (SNR) defined by the ratio of the average signal value to the standard deviation of the signal and the contrast‐to‐noise ratio (CNR) defined by the absolute difference in the average signal between two lesions divided by the average standard deviation of two lesions. To investigate the difference between the denoised and referenced values, the root‐mean‐square error (RMSE) was calculated as follows:

(6) $$\text{RMSE}\left(\text{E}\right)=\sqrt{\frac{\sum_{\text{i}=1}^{\text{N}}{\left[{\overset{¯}{\text{V}}}_{1}\left(\text{E}\right)-{\text{V}}_{\text{i}}^{\text{F}}\left(\text{E}\right)\right]}^{2}}{\text{N}}}$$

where N denotes the total number of pixels in the image, ${\overset{¯}{\text{V}}}_{1}\left(\text{E}\right)$ is the CT number of the averaged VMI (i.e. 10 ND) in the i^th^ pixel, and ${\text{V}}_{\text{i}}^{\text{F}}\left(\text{E}\right)$ is the CT number of the final denoised VMI obtained from one DECT scan (i.e. ND) in the i^th^ pixel.

## 3 RESULTS

**Figure 1:**
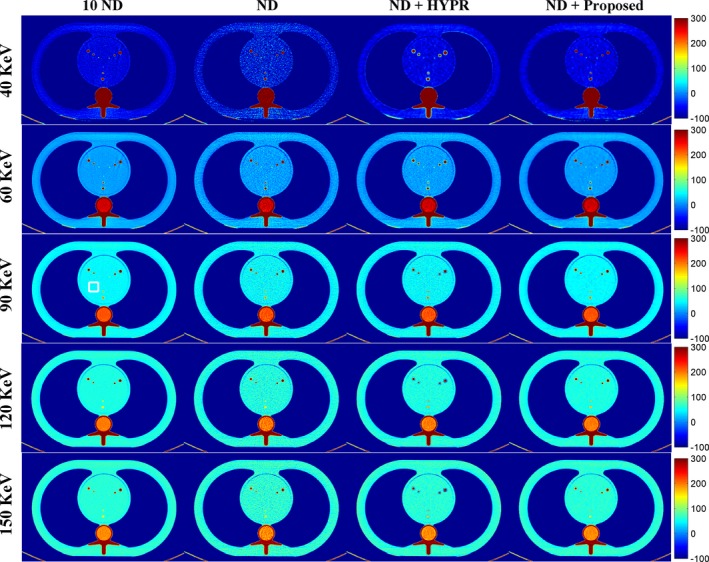
Dual‐energy computed tomography‐derived virtual monoenergetic images of the anthropomorphic cardiac phantom (window settings: level 100 HU, width 200 HU). From top to bottom: 40 keV; 60 keV; 90 keV; 120 keV; 150 keV. From left to right: 10 normal‐dose (ND); ND; ND denoised by the highly constrained backprojection method; ND denoised by the proposed method.

**Figure 2:**
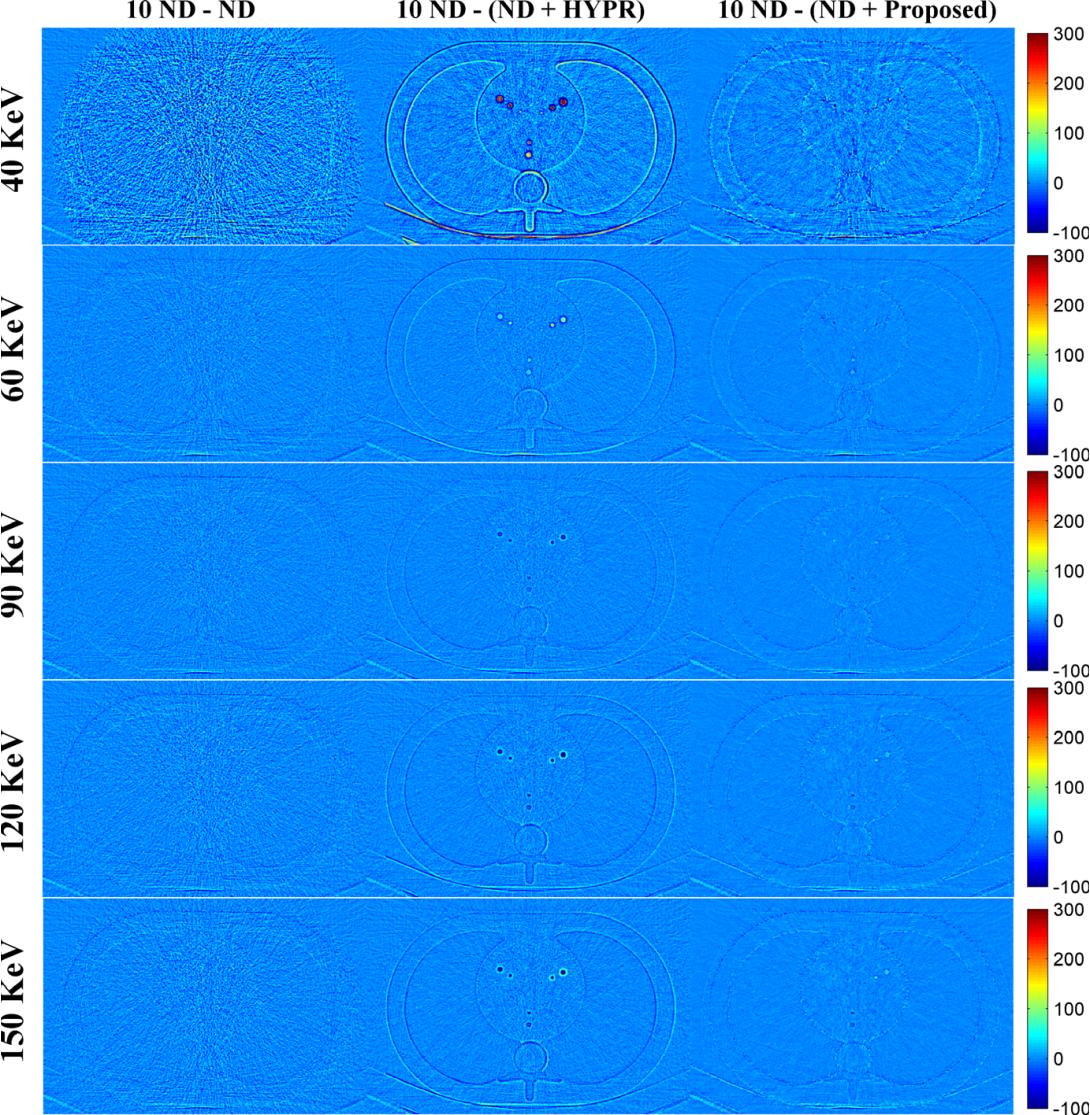
Difference between the 10 normal‐dose (ND) and the results of denoised ND (Fig. 1). From top to bottom: 40 keV; 60 keV; 90 keV; 120 keV; 150 keV. From left to right: 10 ND minus ND; 10 ND minus ND denoised by the highly constrained backprojection method; 10 ND minus ND denoised by the proposed method.

**Figure 3:**
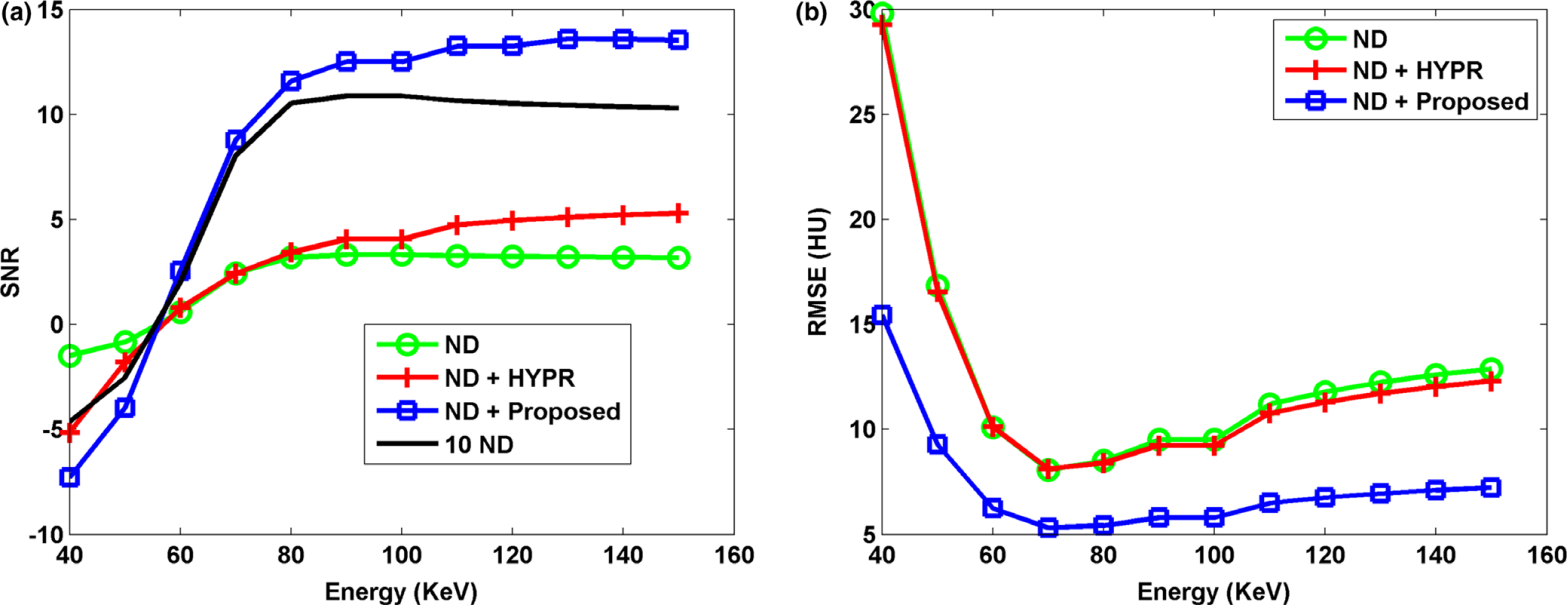
The (a) signal‐to‐noise ratio (SNR) and (b) root‐mean‐square error (RMSE) of various virtual monoenergetic images at different energy levels (40 to 150 keV). The SNR was calculated with a region of interest (see white square shown in Fig. 1). The RMSE was calculated between the 10 normal‐dose (ND) and the results of denoised ND.

**Figure 4:**
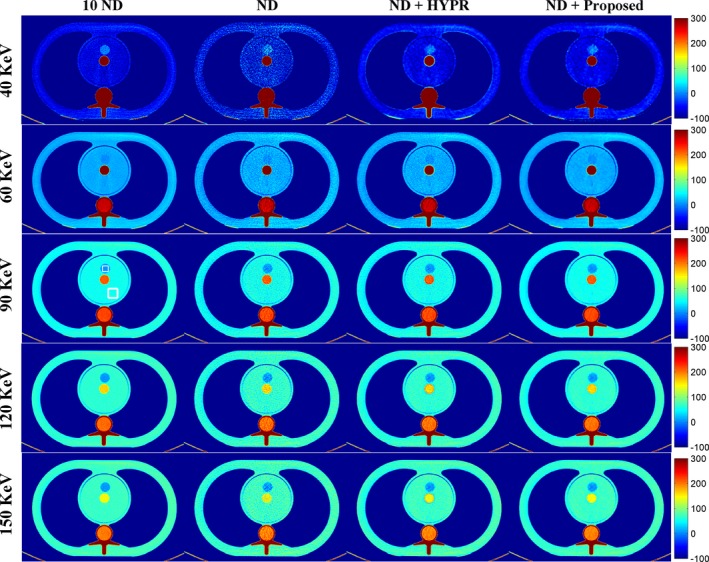
Dual‐energy computed tomography‐derived virtual monoenergetic images of the anthropomorphic cardiac phantom (window settings: level 100 HU, width 200 HU). From top to bottom: 40 keV; 60 keV; 90 keV; 120 keV; 150 keV. From left to right: 10 normal‐dose (ND); ND; ND denoised by the highly constrained backprojection method; ND denoised by the proposed method.

**Figure 5:**
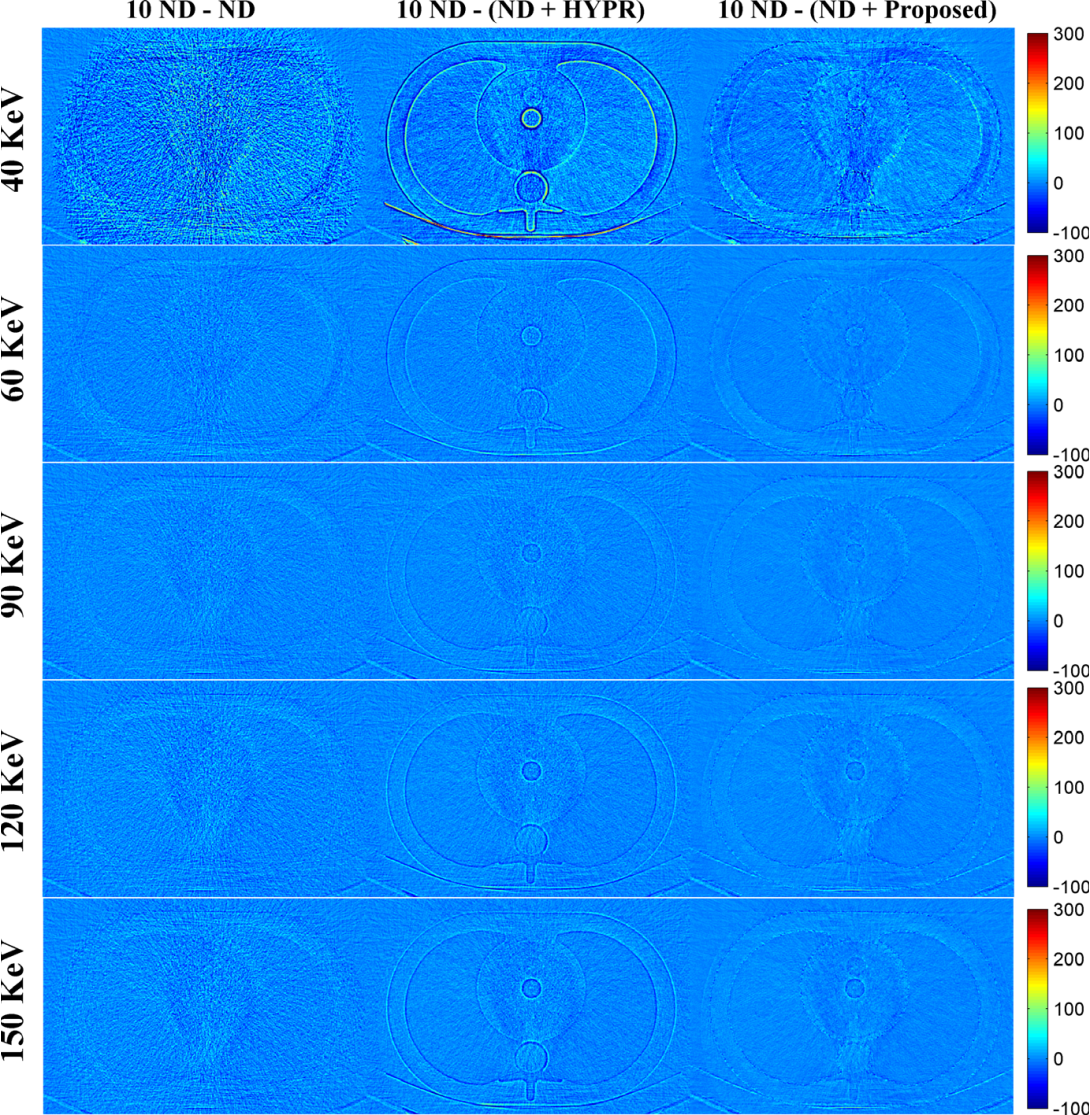
Difference between the 10 normal‐dose (ND) and the results of denoised ND (Fig. 4). From top to bottom: 40 keV; 60 keV; 90 keV; 120 keV; 150 keV. From left to right: 10 ND minus ND; 10 ND minus ND denoised by the highly constrained backprojection method; 10 ND minus ND denoised by the proposed method.

**Figure 6:**
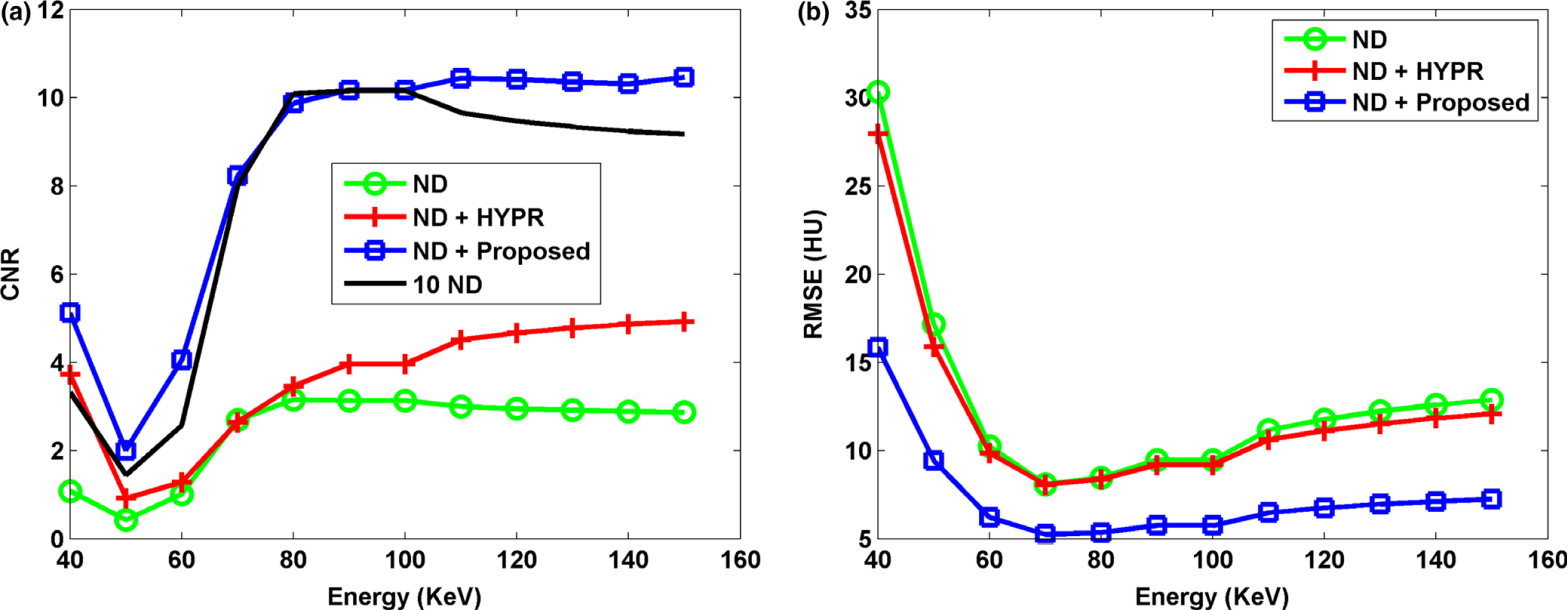
The (a) contrast‐to‐noise ratio (CNR) and (b) root‐mean‐square error (RMSE) of various virtual monoenergetic images at different energy levels (40 to 150 keV). The CNR was calculated with two regions of interest (see white square shown in Fig. 4). The RMSE was calculated between the 10 normal‐dose (ND) and the results of denoised ND.

Figure 1 shows the DECT‐derived VMIs of the anthropomorphic cardiac phantom for the 10 ND, the ND, the ND + HYPR method, and the ND + Proposed method. Compared to the vendor software (i.e. ND), both HYPR and the proposed method can effectively reduce image noise. However, the proposed method provides superior noise reduction, especially in low‐keV VMIs. Figure 2 shows the difference in VMIs between the 10 ND and the other three results (i.e. ND, ND + HYPR and ND + Proposed). It is clear that the loss of intensity in edges and small lesions can be greatly reduced using the proposed method compared to HYPR. Moreover, the objective measures (i.e. SNR and RMSE) shown in Fig. 3 show that the proposed method outperforms HYPR. Note that Fig. 3(a) shows the SNR of the tissue‐equivalent solid material (see white square shown in Fig. 1). Figures 4, 5, 6 are the same as Figs. 1, 2, 3, respectively, but are obtained from a different axial slice. Similar findings can be observed. Note that Fig. 6(a) shows the CNR between the water‐ and tissue‐equivalent solid materials (see white square shown in Fig. 4).

**Figure 7:**
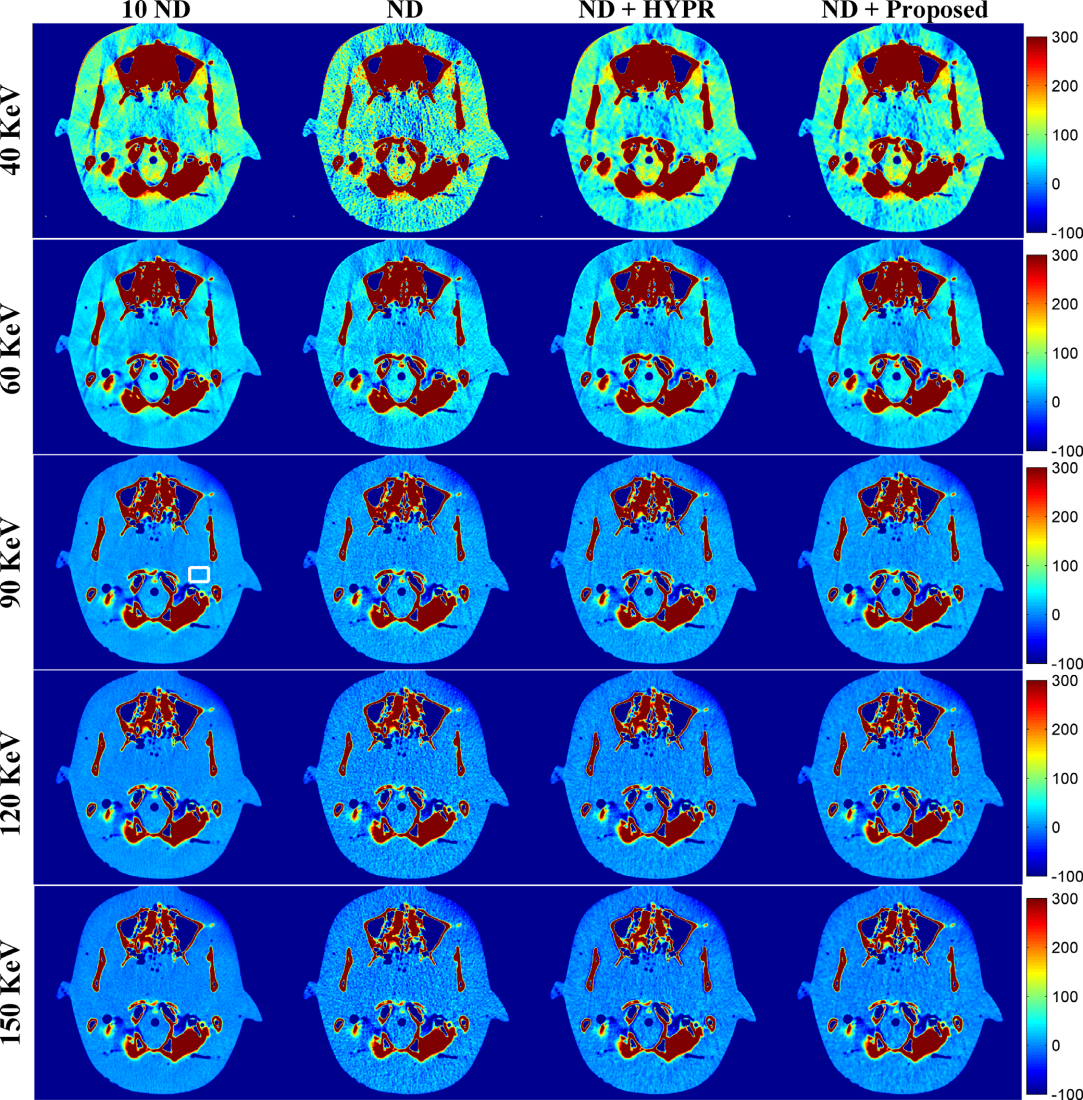
Dual‐energy computed tomography‐derived virtual monoenergetic images of the anthropomorphic brain phantom (window settings: level 100 HU, width 200 HU). From top to bottom: 40 keV; 60 keV; 90 keV; 120 keV; 150 keV. From left to right: 10 normal‐dose (ND); ND; ND denoised by the highly constrained backprojection method; ND denoised by the proposed method.

**Figure 8:**
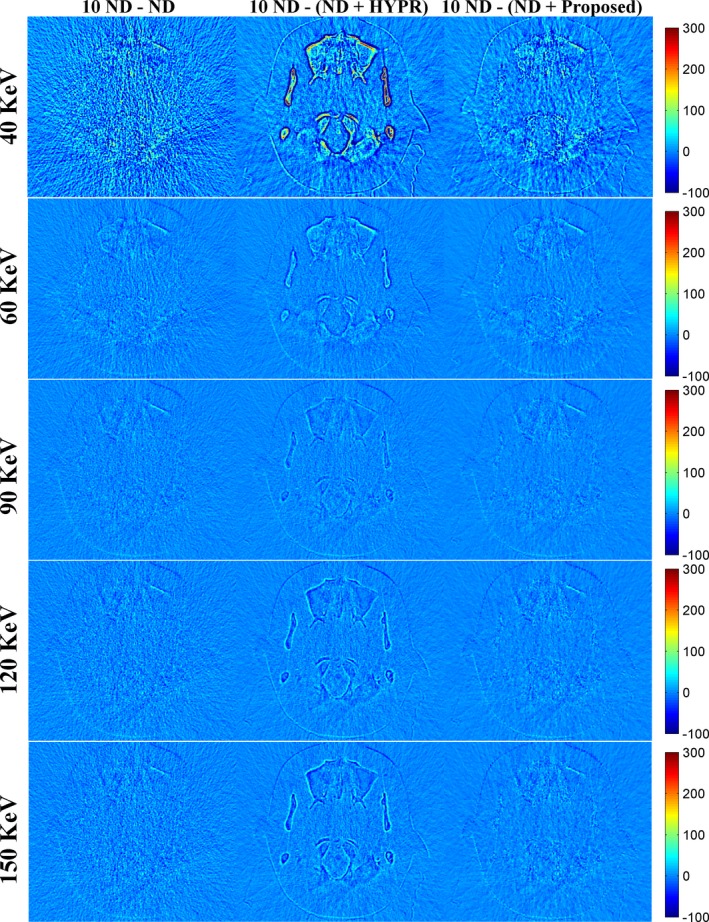
Difference between the 10 normal‐dose (ND) and the results of denoised ND (Fig. 7). From top to bottom: 40 keV; 60 keV; 90 keV; 120 keV; 150 keV. From left to right: 10 ND minus ND; 10 ND minus ND denoised by the highly constrained backprojection method; 10 ND minus ND denoised by the proposed method.

**Figure 9:**
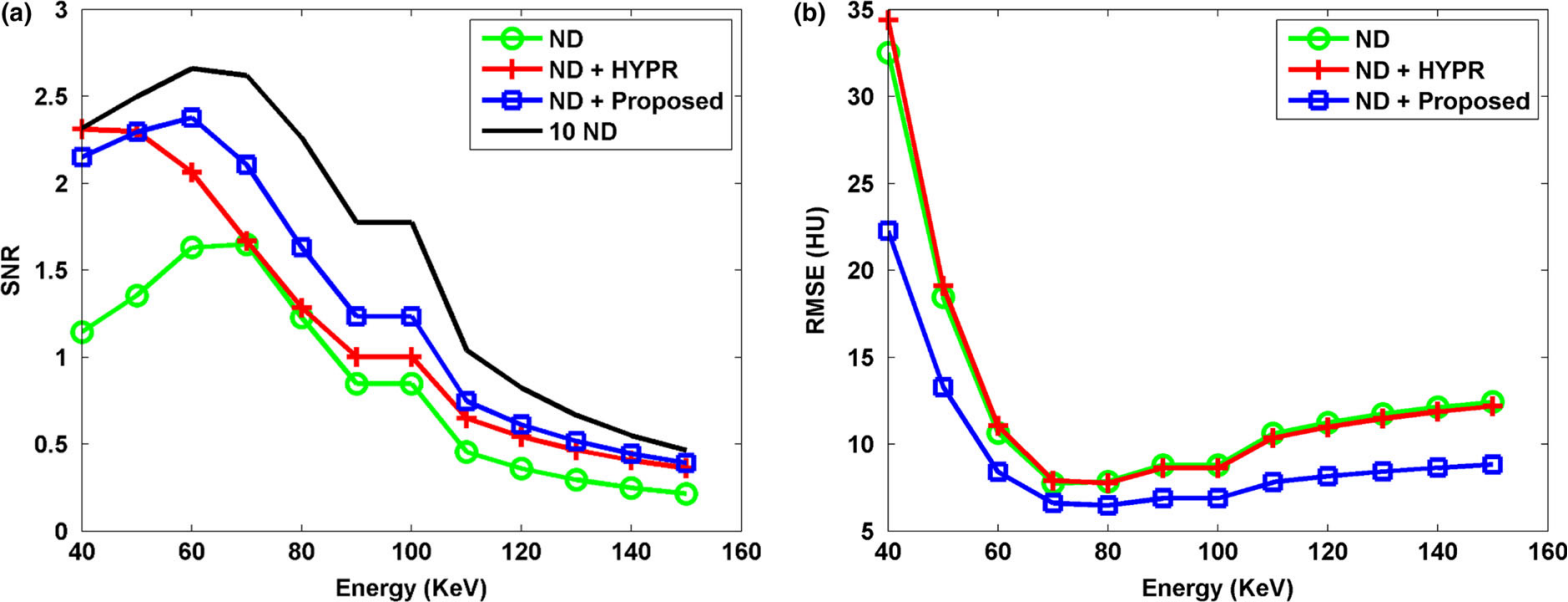
The (a) signal‐to‐noise ratio (SNR) and (b) root‐mean‐square error (RMSE) of various virtual monoenergetic images at different energy levels (40 to 150 keV). The SNR was calculated with a region of interest (see white square shown in Fig. 7). The RMSE was calculated between the 10 normal‐dose (ND) and the results of denoised ND.

To further evaluate the performance of the proposed method, a second phantom study was conducted. Figure 7 shows the DECT‐derived VMIs of the anthropomorphic brain phantom for the 10 ND, the ND, the ND + HYPR method, and the ND + Proposed method. Both HYPR and the proposed method provided image quality better than the vendor software (i.e. ND). Figure 8 shows the difference in VMIs between the 10 ND and the other three results (i.e. ND, ND + HYPR and ND + Proposed). Compared to HYPR, the proposed method has better edge‐preserving performance, especially in low‐keV VMIs. As shown in Fig. 9, the proposed method was superior to HYPR in terms of SNR and RMSE. However, we noticed that the improvement seems limited.

## 4 DISCUSSION

In this study, we propose a two‐step noise reduction method for DECT‐derived VMIs. We modified HYPR^17^, ^18^ by replacing the uniform square convolution kernel with the Wiener filter. To maintain the edge‐preserving smoothing property, we used the results obtained from the Wiener filter with two different window sizes (i.e. 3 × 3 and 7 × 7). Furthermore, we show that GTF^22^ applied to HYPR‐processed VMIs could further improve the image quality of VMIs. Overall, the proposed method has better results than the original HYPR. In particular, the proposed method can reduce not only image noise [Fig. S1(a)] but also edge blurring and the loss of intensity in small lesions for low‐energy (e.g. 40 keV) VMIs [Figs. S1(b) and S1(c)]. The spatial resolution^26^ of VMIs denoised by the proposed method was similar to that of undenoised VMIs (i.e. ND), but the spatial resolution of VMIs was slightly deteriorated by the original HYPR method [Fig. S1(d)]. Although the proposed noise reduction technique uses both the modified HYPR and GTF, these two methods can be used independently. We used the two‐step approach because using either the modified HYPR or GTF provided limited improvement (data not shown).

Despite promising results obtained in this study, we note several issues in the present method. First, the results obtained from two anthropomorphic phantom studies may not be sufficient. Low‐dose data and real patient data should be used to validate the performance of the proposed method. Second, in the case of the anthropomorphic brain phantom, the proposed method provided only moderate improvement. One possible reason is that the GTF method with 40 repetitions seems insufficient for the brain phantom (Figs. 7, 8, 9). The selection of optimal repetitions was not investigated, but will be studied in our future work. Third, we only compared the proposed method with the original HYPR. Other image denoising methods, including the time‐intensity profile similarity bilateral filter,^27^ the partial temporal nonlocal means filter,^28^ and the k‐means clustering guided bilateral filter,^29^ can be used to reduce noise in VMIs. Because these methods have many parameters that need to be optimized, we did not implement these approaches in this study.

In this study, the modified HYPR used composite image obtained from the sum of 12 energy levels (i.e. 40 to 150 keV in 10 keV intervals). We found that the composite image obtained from the sum of six energy levels can provide similar results (data not shown). In other words, increasing the number of energy levels may not improve the performance of the proposed method. One possible reason is that VMIs obtained from neighbouring energy levels have similar image statistical properties. As a result, the advantage of increasing the number of energy levels may be negligible. However, further improvements may be made in the following ways. First, the aforementioned methods^27^, ^28^, ^29^ may be combined either with GTF or with the modified HYPR. Second, the vendor's iterative reconstruction software can be used to produce high‐quality DECT images which may improve the reconstruction of VMIs. Third, DECT‐derived VMIs reconstructed using iterative image‐domain decomposition methods^30^, ^31^ may have improved image quality.

## 5 CONCLUSION

VMIs derived from DECT have shown encouraging results for a broad clinical application. However, the image noise of the VMI was high at low and high energies. We have demonstrated that the proposed two‐step image denoising method can reduce image noise in different energy‐level VMIs. Moreover, the proposed image denoising method can reduce edge blurring and alleviate the loss of intensity in small lesions.

## CONFLICTS OF INTEREST

The authors have no relevant conflicts of interest to disclose.

## Supporting information


**Fig. S1**. (a) Noise of various virtual monoenergetic images (VMIs) at different energy levels (40 to 150 keV) and horizontal profiles crossing three different sized circles for (b) 40‐keV and (c) 50‐keV VMIs. (d) The modulation transfer function curves of various VMIs at 40 keV. The noise was calculated with a region of interest (see white square shown in Fig. 1)
