# Supplementary figures and images for: Noise reduction in dual‐energy computed tomography virtual monoenergetic imaging
Source: J Appl Clin Med Phys. 2019 Aug 7;20(9):104–13. doi: 10.1002/acm2.12694 (PMC6753738; doi:10.1002/acm2.12694)

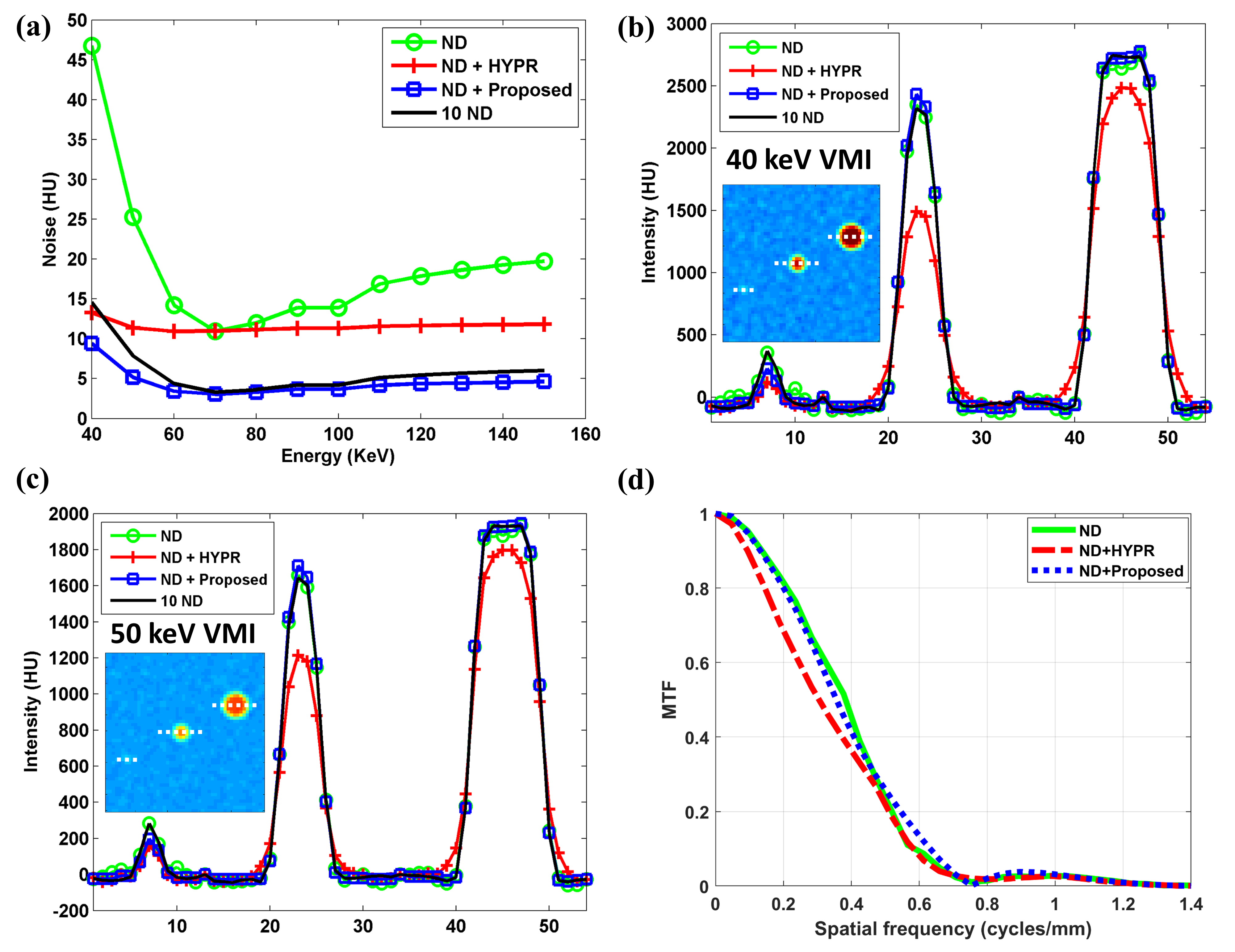

Supplement: Supplementary file 1 — Fig. S1. (a) Noise of various virtual monoenergetic images (VMIs) at different energy levels (40 to 150 keV) and horizontal profiles crossing three different sized circles for (b) 40‐keV and (c) 50‐keV VMIs. (d) The modulation transfer function curves of various VMIs at 40 keV. The noise was calculated with a region of interest (see white square shown in Fig. 1) [file ACM2-20-104-s001.tif]
